# CLINICAL APPLICATION OF A DRILL GUIDE TEMPLATE FOR PEDICLE SCREW PLACEMENT IN SEVERE SCOLIOSIS

**DOI:** 10.1590/1413-785220172502138828

**Published:** 2017

**Authors:** Xin Li, Yaoshen Zhang, Qiang Zhang, Changsong Zhao, Kun Liu

**Affiliations:** 1 Department of Orthopedics, Beijing Ditan Hospital, Capital Medical University, Beijing, China; 2 Beijing Chaoyang Hospital, Capital Medical University, Beijing, China; 3 The Hospital of Shunyi District Beijing, Beijing, China

**Keywords:** Scoliosis, Pedicle screws, Surgery, computer-assisted/methods, Imaging, three-dimensional, Prosthesis design, Printing, three-dimensional

## Abstract

**Objective:**

To evaluate the accuracy and the effect of drill guide template for pedicle screw placement in severe scoliosis.

**Method:**

Eight patients with rigid scoliosis were enrolled, five males and three females, ranging from nine to 23 years old. A three-dimensional CT scan of the spine was performed and saved as a DICOM file type. The multi-level template was designed by Mimics software and manufactured according to the part of the most severe deformity. The drill template was placed on the corresponding vertebral surface. Pedicle screws were carefully inserted across the trajectory of the template. Postoperatively, the positions of the pedicle screws were evaluated by CT scan and graded for validation.

**Results:**

No spinal cord injury or nerve damage occurred. All patients had satisfactory outcomes. The abnormalities and the measures observed during operation were the same as those found in the preoperative period. The position of the pedicle screws was accurate, according to the postoperative X-ray and CT scan. The rate of scoliosis correction was 60%. Compared with controls, surgery time, blood loss and radiation were significantly lower.

**Conclusion:**

With the application of multi-level template, the placement of pedicle screws shows high accuracy in scoliosis with shorter surgical time, less blood loss and less radiation exposure. ***Level of Evidence III, Retrospective Comparative Study.***

## RESUMO

**Objetivo:**

Avaliar a precisão e o efeito da matriz de guia de broca para colocação de parafuso pedicular em escoliose grave.

**Método:**

Oito pacientes com escoliose rígida foram selecionados, sendo cinco homens e três mulheres na faixa etária de nove a 23 anos. Foi realizada TC tridimensional da coluna, gravada no formato DICOM. A matriz multinível foi desenhada pelo software Mimics e fabricada de acordo com a parte da deformidade mais grave. A matriz da broca foi colocada na superfície vertebral correspondente. Os parafusos pediculares foram cuidadosamente inseridos ao longo da trajetória da matriz. No pós-operatório, as posições dos parafusos pediculares foram avaliadas por TC e classificadas para validação.

**Resultados:**

Não ocorreu nenhuma lesão da medula espinhal nem lesão de nervos. Todos os pacientes tiveram desfechos satisfatórios. As anormalidades e as medidas observadas durante a operação foram as mesmas encontradas no pré-operatório. A posição dos parafusos pediculares foi precisa, de acordo com a radiografia e a TC pós-operatórias. A taxa de correção de escoliose foi de 60%. Em comparação com os controles, o tempo de cirurgia, a perda de sangue e a radiação foram significantemente menores.

**Conclusão:**

Com a aplicação da matriz multinível, a colocação dos parafusos pediculares mostra alta precisão na escoliose, com menor tempo cirúrgico, menos perda de sangue e menor exposição à radiação. Nível de Evidência III, Estudo Retrospectivo Comparativo.

## INTRODUCTION

Computer-aided rapid prototyping (RP) using Mimics medical imaging software can be simulated in a computer-based operation using an operational design and planning scheme which displays the results of the procedure. This method also permits rapid input on a digital machine which can copy the real structure for an entity model and allow the surgeon to simulate and evaluate intraoperative references and guides for positioning and navigation prior to the procedure. Rapid prototyping has been widely applied in the medical field in China and abroad in recent years; this technology is used in the fields of complex fractures, spine and extremity deformities, joint replacement, plastic surgery, cranial and facial tumors, nasal reconstruction, dental implants and prosthesis engineering,^1^ ^-^ ^6^ as well as for planning procedures in clinical medicine. Biological manufacturing provides a more effective solution and means of production. The aim of this study is to investigate the use of a RP technique to construct a pedicle drill template as well as to assess and evaluate the outcomes.

## MATERIALS AND METHODS 

This study was approved by the institutional review board of Beijing Ditan Hospital at Capital Medical University under number 1501/2009. Written informed consent was obtained from all patients prior to enrollment in the study. From June 2006 to October 2009, 8 patients (5 males and 3 females) with scoliosis were evaluated; the participants ranged in age from 9 to 23 (average age: 18). Five subjects had congenital scoliosis and 3 had idiopathic scoliosis; 4 patients underwent a repeat operation. Preoperatively, the average Cobb angle was 91° (70°~125°), and the average kyphosis Cobb angle was 65° (45°~95°). The preoperative Frankel scores were as follows: 1 case with C, 1 case with D, and 6 cases with E, including 1 case with bladder sphincter dysfunction. Five cases had serious thoracolumbar back pain. During the preoperative period, the patients underwent plain film X-rays, MRI scans, and three-dimensional reconstruction of spiral CT. All patients had different degrees of spina bifida, butterfly vertebra, congenital fusion of the ribs, spinal bone ridge, posterior fusion, and severe vertebral rotation. At the same time, 8 severe scoliosis patients who did not undergo spinal modeling were selected at random to comprise the control group.

**Figure 1:**
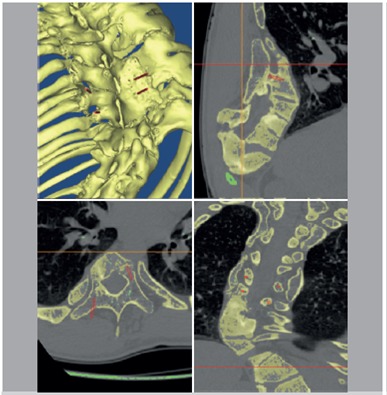
Placement, diameter, and depth direction of the pedicle screw defined using Mimics software with the three-dimensional reconstruction data.

**Figure 2:**
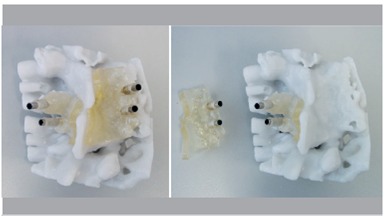
Attaching the pedicle drill navigation template to the back of the vertebrae and rotating the model in each direction to observe the positioning guide hole and accuracy of pedicle placement.

Preoperatively, all the patients underwent full spine enhanced CT scanning with a 1 mm slice thickness. We collected the original CT data and used a DICOM format input computer and Mimics 6.3 software (Materialise N.V., Haasrode, Belgium) for three-dimensional reconstruction of the digital display as well as to measure the spinal data. We used the CT data to perform the operation, reconstruct the surgical region, define placement, diameter, and depth direction of the pedicle screws using the software design of the virtual pin tract, avoid neurovascular organs, and ensure that the pedicle screws designed prior to the procedure were in the pedicle to avoid breaking through the bone. (Figure 1) We identified the best screw trajectory and location based on the RP spine pedicle screw placement in the real model. We also made the drill guide template, designed the surgical plan, simulated the procedure in models, and ensured that pedicle screw placement was more intuitive and accurate. We also attached the three-dimensional reconstruction of the vertebral model template to the back of the vertebrae and rotated the model in each direction, observing the positioning guide hole and the accuracy of pedicle placement. (Figure 2)

**Figure 3:**
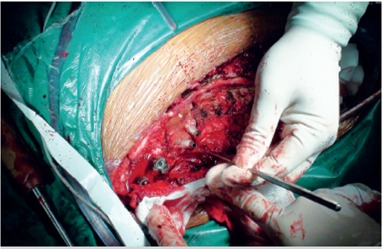
The intraoperative view was the same as the three-dimensional reconstruction and rapid prototyping model. The navigation template and deformity were a close fit. Needle passage using the navigation template was correct and safe.

The rapid prototyping guide template was managed by low temperature plasma sterilizer. All patients received general anesthesia prior to surgery to correct the posterior deformity utilizing the pedicle screw fixation technique, intraoperative findings, and preoperative three-dimensional reconstruction. The RP model showed that the results were consistent and that the navigation template and deformity were a close fit. The preoperative design permitted successful correction of the deformity via placement of pedicle screws, and intraoperative fluoroscopy showed that the pedicle implant was well-positioned and that the template fit well. (Figure 3) There were 5 cases of complete vertebral osteotomy and 3 cases of simple internal fixation. Excision of the ribs and vertebrae and bone allograft were routinely performed. Four cases involved autologous blood transfusion. Surgical time, volume of blood lost, and fluoroscopy frequency data were recorded.

## RESULTS

All 8 patients underwent digital reconstruction of the skeleton, which allowed us to observe a case of severe congenital scoliosis from any direction by constructing a spinal model. This model in turn allowed us to measure the extent of the deformity, understand the range and shape of the spina bifida, and to understand the scope and shape divided by intraspinal osseous lesions as well as the relationship between the spinal cord and the configuration of the spinal canal. A RP spine model design for surgery was created for all cases. The intraoperative findings and preoperative 3D reconstruction results were completely consistent. Eight patients were scheduled for the procedure; in these cases, we could clearly identify the anatomical structure of the spinal deformity and pedicle screw positioning was adequate. All cases healed well without wound infection, spinal cord injury, cerebrospinal fluid leakage, or complications such as pneumothorax, and the patients obtained good postoperative results. Additionally, the findings from the surgical procedure and the preoperative three-dimensional reconstruction of the digital model for abnormal findings and measurement results were completely consistent. Postoperative X-ray and CT demonstrated that adequate positioning of the pedicle screws. The scoliosis correction rate was 60%, and the average surgical time was 186 min. Average blood loss was 460 ml, and C-arm fluoroscopy was used 4 times. Using guide navigation in the patient group, the average surgical time was 225 minutes, average blood loss was 550 ml, and fluoroscopy was used approximately 30 times.

## DISCUSSION

Modern computer technology allowed the development of a digital model of the human skeleton and RP technology in the late twentieth century, providing new technologies for use in clinical medicine. Human CT scan data are processed with a computer to create three-dimensional digital images of structures in the human body, including lesion sites, and DICOM data are acquired using a computer. This technique also uses CAD, Mimics 6.3, and 3D View 3.5 software for three-dimensional reconstruction and measurement display. The laser stacking method has been used for RP in plastic surgery.^1^ ^-^ ^6^ ^)^ The field of orthopedics has seen revolutionary developments, and this methodology is currently used in orthopedic reconstructions. Additionally, this positioning technology has been widely applied in more invasive orthopedics operations to improve accuracy.^2^ ^,^ ^6^ ^-^ ^8^ As the difficulty of these operations has increased, doctors no longer depend on the patient's medical history alone; instead, images are required for the surgeon to determine the surgical plan. Consequently, surgeons should be able to use surgical planning software, establish a three-dimensional anatomical model of the target site, and design the procedure on the computer screen in order to achieve the best surgical plan. These skills will be required of clinicians in the twenty-first century.^6^

The anatomy of the spine is very complex, and the complex morphologic structure of scoliosis has several associated variations, including kyphosis, vertebral rotation, wedge, spondylolisthesis deformities, spinal deformities, severe thoracic spinal deformation with a small, irregular vertebral arch root, and deformation of the spinal cord, nerve root, adjacent lung, esophagus, aorta, inferior vena cava, and other important organs and large vessels. Consequently, congenital scoliosis with pedicle screw placement has presented a challenge to surgeons for some time, particularly in patients with spina bifida, butterfly vertebrae, and congenital posterior fusion. Such patients are treated with an anterior approach, but the posterior or anterior and posterior approach for correction can encounter many difficulties. Accurate understanding of the specific malformations well in advance of surgical planning and the procedure itself is essential.^2^ However, it can be difficult to fully and clearly view the deformities in specific circumstances due to scoliosis, vertebral rotation, and overlapping morphological variation in images, even with the use of conventional X-ray plain films, CT, or two-dimensional MRI tomography. Even with preoperative three-dimensional reconstruction, it can be difficult to acquire a visible and tangible three-dimensional view and spine model. However, RP of the spine uses a human skeleton as the reconstruction of the spine. It is the same size as the patient's bones but does not present any trauma when used for surgical planning and simulation and the procedure. In cases of severe spine deformity, the spinal and morphology and anatomical landmarks can undergo tremendous changes and be difficult to recognize. Scoliosis can substantially alter the vertebral morphology, and judging the screw entrance point can be a major challenge in clinical work. The navigation guide plate was recently developed based on the digital spine model in pedicle screw placement, and may present a solution for this challenge.^3^ ^,^ ^4^ ^,^ ^9^ ^,^ ^10^ Preoperatively, computerized surgical plans have been used to select the correct screw entry point and suitable pedicle screws. Additionally, the RP spine model has been used to produce the pedicle navigation guide and to avoid surgical errors, save time, reduce the use of intraoperative fluoroscopy, and alleviate patient pain.

The free hand technique is still the most commonly used clinical placement method, intraoperative C-arm fluoroscopy, and computer-aided navigation. The acts technique is still the most commonly used clinical placement method.^3^ ^,^ ^4^ ^,^ ^11^ Generally, when the surgeon has sufficient experience in spinal surgery, the spinal deformity is not serious and the anatomic landmarks are clearly defined, the accuracy of freehand pedicle screw placement can be assured using a pedicle probe. Some studies have reported that the rate of pedicular cortical perforation ranges from 6.2% to 72.4%; rates of neural, vascular and visceral injuries as well as other complications range from 0% to 0.9%.^12^ ^-^ ^17^ Intraoperative C-arm fluoroscopic X-ray can increase the accuracy of pedicle screw placement, but extended surgical time, increased intraoperative fluoroscopy, and repeated adjustment of pedicle screw placement direction can also cause screw loosening or failure. Complex vertebral malformations make it difficult to place the screw in the correct position; blind placement can cause injuries to the spinal cord, nerves, blood vessels, and adjacent viscera. Computer-assisted navigation technology can be used to increase the accuracy of pedicle screw placement. However, complex vertebral malformations make it difficult to place screws in the correct position, and the procedure is complicated, time-consuming and laborious. Additionally, navigation devices are expensive, accuracy is low, there is a high learning curve, and there are other shortcomings; furthermore, they have not been used in Chinese hospitals.

As pedicle screw fixation technology has developed and matured, these techniques have been widely used in the treatment of adult spinal trauma and degeneration, orthopedic surgery, and tumor resection.^1^ ^-^ ^6^ Severe vertebral rotation may be present in these cases, or patients may require multiple operations, and surgeons often have doubts about how to replace the pedicle screw because the allowable deviation range is small, especially in the thoracic spine, and risk increases. In the past, these surgeries relied on the surgeon's experience and repeated C-arm X-rays to adjust the position of the pedicle screw; this procedure largely depends on the surgeon's experience and luck. If all goes well, the screw can be inserted smoothly, but in some cases a loose screw may need repeated adjustments, may not be placed, or may cause injury. In severe cases, mistakes can cause disability or death, and the risk is high. Spinal surgeons are paying greater attention to the accuracy of thoracic pedicle screw placement and safety.^11^ ^-^ ^15^ To improve accuracy of pedicle screw placement, we used RP technology to making a drill guide template for pedicle navigation, which made placement of pedicle screw as a safe and feasible method for congenital scoliosis. Reverse engineering and RP technology^15^ were the keys of design. We used reverse engineering software to extract the lamina surface from the posterior vertebral anatomy, then established optimal pedicle screws trajectory. The drill guide templates were designed according to these data, so that its placement can be consistent with the posterior vertebral. And RP technology can produce the dill guide template for personalized individuals. In the operation, template and corresponding thoracic posterior bony structure fit well. Made position and channels along the drill of template can be ensure each screw placed correctly. The fluoroscopy and postoperative CT confirmed adequate pedicle screw placement.

The template was made to fit the bone closely according to the anatomical structure, allowing more stable fixation; in theory, greater contact between the template and bone provides greater stability. However, it is important that the template not be too large or it may reduce the motion of the intervertebral spinal segment. In our experience, it is best to not manipulate more than two segments of the vertebral plate and facet joint. A 3-5 centimeter guide sleeve is advisable; if the sleeve is too short, accuracy will also be affected. During the procedure, the spinous process, laminectomy, and facet joint are fully exposed so that the guide plate and corresponding segment of the spinal bone can fit fully. Next, the cannula is used to open, drill, sound, and remove the guide; detection of the pedicle bone is complete at this time, permitting evaluation of tapping and the pedicle screw. Additionally, the navigation template for screw preparation (preferably using a drill or drills) can reduce shaking with the borehole. This allows the screw channel to be prepared as exactly as possible according to the direction of the guiding channel, and the surgeon can strive to follow the template design of the navigation positioning. To reduce error and increase the precision of the guide, a CT scan is best in patients prone to spinal operation bed frame defects because CT is closer to the patient's intraoperative position and is as far as possible from postural changes that may influence the guide's accuracy. To ensure that the operation is not in danger of failing, pedicle screw placement is completed with the assistance of a C-arm X-ray check.

In short, RP guide navigation technology can quickly produce the desired prototype without requiring substantial experience with the procedure, and can also simulate the surgical process and possible problems, allowing prior consideration of remediative and preventive measures. The use of an operation navigation guide plate in severe scoliosis procedures improves safety, shortens surgical time, reduces surgical trauma, and reduces bleeding as well as intraoperative fluoroscopy. It is safe, accurate, and minimally invasive.^3^ ^,^ ^4^ ^,^ ^15^ In addition to ensuring speed and accuracy (and in turn, reducing patient suffering and postoperative complications), this method can relieve the economic burden on patients and produce positive economic and social benefits.

## CONCLUSION

The RP pedicle drill template is a new method for precise insertion of pedicle screws in operations. This method is highly accurate and safe in scoliosis spinal procedures. Utilizing the pedicle drill template navigation procedure is simple, and does not require extensive specific experience on the part of the surgeon. This method can shorten surgical time and reduce blood loss, and the surgeon's exposure to radiation can be reduced or avoided with this technique.

## ACKNOWLEDGMENTS

This study was supported by grants from Basic clinical research cooperation projects of CCMU(16JL08).

## References

[B1] Wang J, Yin QS, Xia H (2011). Computer-aided design-rapid prototype used in trans-oralpharyngeal atlantoaxial reduction plating. Chin J Orthop Trauma.

[B2] Hu Y, Yuan ZS, Spiker WR, Albert TJ, Dong WX, Xie H (2013). Deviation analysis of C2 translaminar screw placement assisted by a novel rapid prototyping drill template: a cadaveric study. Eur Spine J.

[B3] Ma T, Xu YQ, Cheng YB, Jiang MY, Xu XM, Xie L (2012). A novel computer-assisted drill guide template for thoracic pedicle screw placement: a cadaveric study. Arch Orthop Trauma Surg.

[B4] Lu S, Zhang YZ, Wang Z, Shi JH, Chen YB, Xu XM (2012). Accuracy and efficacy of thoracic pedicle screws in scoliosis with patient-specific drill template. Med Biol Eng Comput.

[B5] Müller A, Krishnan KG, Uhl E, Mast G (2003). The application of rapid prototyping techniques in cranial reconstruction and preoperative planning in neurosurgery. J Craniofac Surg.

[B6] D'Urso PS, Barker TM, Earwaker WJ, Bruce LJ, Atkinson RL, Lanigan MW (1999). Stereolithographic biomodelling in cranio-maxillofacial surgery: a prospective trial. J Craniomaxillofac Surg.

[B7] Pei G, Zhang Y (2009). Digital orthopaedics.

[B8] Zhang Y, Lu S, Zhao J (2011). Digital technologu used in orthopaedic surgery. Chin J Orthop Trauma.

[B9] Di Silvestre M, Parisini P, Lolli F, Bakaloudis G (2007). Complications of thoracic pedicle screws in scoliosis treatment. Spine (Phila Pa 1976).

[B10] Owen BD, Christensen GE, Reinhardt JM, Ryken TC (2007). Rapid prototypepatient-specific drill template for cervical pedicle screw placement. Comput Aided Surg.

[B11] Hong-Xun S, Wei L, Zhensheng M (2008). Application of rapid prototyping in the treatment of severe congenital spinal deformities. Chinese Journal of Spine and Spinal Cord.

[B12] Xu R, Ebraheim NA, Ou Y, Yeasting RA (1998). Anatomic considerations of pedicle screw placement in the thoracic spine. Roy-Camille technique versus open-lamina technique. Spine (Phila Pa 1976).

[B13] Ya S, Hou S, Wei X (2003). The Imaging and clinical character of adolescent thoracic pedicle. Orthop J China.

[B14] Lu S, Xu Y, Li Y (2008). A new digital template as navigation in spinal pedicle instrumentation. Chin J Orthop Trauma.

[B15] Oertel MF, Hobart J, Stein M, Schreiber V, Scharbrodt W (2011). Clinical and methodological precision of spinal navigation assisted by 3D intraoperative O-arm radiographic imaging. J Neurosurg Spine.

[B16] Wu ZX, Huang LY, Sang HX, Ma ZS, Wan SY, Cui G (2011). Accuracy and safety assessment of pedicle screw placement using the rapid prototyping technique in severe congenital scoliosis. Spinal Disord Tech.

[B17] Carbone JJ, Tortolani PJ, Quartararo LG (2003). Fluoroscopically assisted pedicle screw fixation for thoracic and thoracolumbar injuries: technique and short-term complications. Spine (Phila Pa 1976).

